# Extracellular vesicles from methicillin resistant *Staphylococcus aureus* stimulate proinflammatory cytokine production and trigger IgE-mediated hypersensitivity

**DOI:** 10.1080/22221751.2021.1991239

**Published:** 2021-10-08

**Authors:** Krisana Asano, Shouhei Hirose, Kouji Narita, Phawinee Subsomwong, Noriaki Kawai, Rojana Sukchawalit, Akio Nakane

**Affiliations:** a Department of Microbiology and Immunology, Hirosaki University Graduate School of Medicine, Hirosaki, Aomori, Japan; b Department of Biopolymer and Health Science, Hirosaki University Graduate School of Medicine, Hirosaki, Aomori, Japan; c Institute for Animal Experimentation, Hirosaki University Graduate School of Medicine, Hirosaki, Aomori, Japan; d Laboratory of Biotechnology, Chulabhorn Research Institute, Lak Si, Bangkok, Thailand

**Keywords:** *Staphylococcus aureus*, methicillin resistance, membrane vesicles, inflammatory stimulation, IgE-mediated hypersensitivity

## Abstract

Extracellular vesicles (EVs) released from bacteria are enclosed particles carrying biological active molecules. They have been shown to play a role in bacterial communications and delivery of virulence factors to the host cells. *Staphylococcus aureus* is an opportunistic pathogen causing a variety of infections ranging from impetigo to septicaemia. The EVs released from *S. aureus* have a high potential to be used for vaccine development against *S. aureus* infections. However, it is important to clearly understand the impact of SaEVs on the host’s immune response. Our study demonstrated that purified EVs from a clinical isolated methicillin-resistant *S. aureus* (SaEVs) significantly stimulated proinflammatory cytokine production in mouse immune cells and induced host cell death. An impairment of cytokine production in the Toll-like receptor (TLR)-silenced macrophages suggested that SaEVs stimulate proinflammatory response via TLRs 2, 4 and 9. In mouse infection model, the results demonstrated that SaEV immunization did not provide protective effect. In contrast, all SaEV-immunized mice died within Day 1 after methicillin-resistant *S. aureus* (MRSA) infection. After MRSA infection for 3 h, the production of IL-6, TNF-α and IL-17 in the spleen of SaEV-immunized mice was significantly higher than that of control mice. On Day 5 after the second immunization, total IgE in the serum was significantly enhanced, and a high titre of Th2-related cytokines was remarkably induced after *ex vivo* stimulation of the spleen cells with SaEVs. These results suggested that MRSA-derived EVs act as an immunostimulant that induces inflammatory response and IgE-mediated hypersensitivity after MRSA infection.

## Introduction

*Staphylococcus aureus* is a Gram-positive bacterium that is commonly found on human skin and persists in the nasal cavity [1]. This bacterium has evolved mechanisms to survive with host as a commensal microbiota. However, it is also an opportunistic pathogen causing a wide variety of infections as well as life-threatening diseases, especially in the immunocompromised patients. Several virulence factors produced from *S. aureus* can cause severe clinical manifestations including toxic shock syndrome [1]. In particular, methicillin-resistant *S. aureus* (MRSA) has become a worldwide major problem in healthcare facilities and communities because it resists to many common antibiotics [2]. Therefore, a better understanding of *S. aureus*-host interaction is important and could provide useful information for the development of novel strategies for the prevention and successful treatment of MRSA infection. Immunization against *S. aureus* would be beneficial for people at risk for invasive MRSA infections such as the patients with known MRSA colonization, and the patients who are scheduled for surgery or foreign body implantation.

Extracellular vesicles (EVs) released from bacteria are membrane-bound particles containing various biological active molecules such as DNA, RNA, lipids and proteins [3,4]. Although the role of bacterial EVs remains to be elucidated, these particles are considered to play a role in bacterial communication, stimulation of host immune response and delivery of virulence factors to the host cells [3,4]. Based on their enclosed structure, the EVs are able to protect the biological active molecules from the environmental degrading enzymes such as nucleases and proteases [5]. In addition, the surface molecules on EVs offer the ligands that facilitate the interaction to the target cells [5]. Therefore, the EVs are considered to translocate over a long-distance and transfer the biological active molecules to the specific targets. The presence of various antigens within EVs makes these particles become more attractive for clinical applications such as diagnostic tool and vaccine against pathogenic bacteria. To date, the vaccine platform concepts based on bacterial EVs are under development [6].

Several studies have shown that *S. aureus* is capable of secreting EVs during *in vitro* cultures [7–11] and various proteins in these vesicles have been identified [7–10]. In addition, the EVs from *S. aureus* have been shown to induce host cell death [8,9,11] and stimulate inflammatory response [10,11]. However, these effects were dependent on the *S. aureus* isolated strains and host cell types. In the present study, EVs were purified from a clinical isolated MRSA strain 834 (SaEVs) and the effect of SaEVs on the host immune response was examined. Mouse immune cells were treated with SaEVs and the production of proinflammatory cytokines including tumour necrosis factor alpha (TNF-α), interleukin 6 (IL-6) and interferon gamma (IFN-γ) was determined. RNA interference of Toll-like receptors (TLRs) was performed to predict the molecules in SaEVs that stimulate the proinflammatory cytokine production. In addition, the cytotoxic effect of SaEVs was evaluated. Regarding a high potential of SaEVs as a vaccine, we also investigated the protective effect of SaEV-immunization against MRSA infection. Our results demonstrated that IgE-mediated hypersensitivity is induced in the SaEV-immunized mice, and the hyperinflammatory shock in these mice is induced immediately after MRSA infection.

## Materials and methods

### Mice and ethical statement

Female BALB/c mice were purchased from CLEA Japan, Inc., Tokyo, Japan. They were housed under specific pathogen-free conditions with a temperature-controlled room (22 ± 2°C) on a 12-h light–dark cycle at the Institute for Animal Experimentation, Hirosaki University Graduate School of Medicine. They were allowed ad libitum access to tap water and food pellets (CE-2, CLEA Japan). All animal experiments in the present study were conducted in accordance with the Animal Research Ethics Committee, Hirosaki University Graduate School of Medicine, and followed the Guidelines for Animal Experimentation, Hirosaki University (Permit number: M19002). To reduce pain and stress during experiments, mice were euthanized using a mixture of 0.3 mg/kg medetomidine, 4 mg/kg midazolam and 5 mg/kg butorphanol or inhaled isoflurane.

### Bacterial strain and growth conditions

A clinical MRSA isolate, *S. aureus* 834 [12] was cultivated and maintained at 37°C for 16–24 h in tryptic soy broth (BD Bioscience, Sparks, MD) and tryptic soy agar, respectively. The activated bacterial cells were inoculated into Brain Heart Infusion medium (BD Bioscience) and cultured under aerobic condition at 37°C for 8 h. The supernatant was then collected by centrifugation twice at 5000×*g*, 4°C for 20 min, filtrated through 0.45 μm filter (Merck Millipore Ltd., Tullagreen, Ireland) to remove all remaining bacterial cells and kept at −80°C until SaEV purification. For mouse infection model, MRSA 834 were cultured in tryptic soy broth as mentioned above. The bacterial cells were harvested by centrifugation at 5000×*g* for 20 min and washed with phosphate-buffered saline (PBS). After suspension in PBS, the bacterial cells were adjusted spectrophotometrically at 550 nm to be 5 × 10^8^ CFU/mL (OD_550nm_ of 1.0 correlates to 3.3 × 10^8^ CFU/mL).

### Purification of SaEVs

SaEVs in the culture supernatant of MRSA 834 were harvested by ultracentrifugation at 100,000×*g*, 4°C for 90 min using a Himac CP80WX Preparative Ultracentrifuge (HITACHI, Tokyo, Japan) with a P55AT fixed angle rotor. The supernatant was carefully removed, and crude pellet containing SaEVs was washed and suspended in 1 mL of ice-cold PBS (from an initial volume of 4.8 L culture supernatant, the suspended volume is about 1 mL). SaEVs were then purified using an OptiPrep™ density gradient according to the standard protocol [13] with some modifications. Briefly, a discontinuous iodixanol gradient was prepared by diluting a stock solution of OptiPrep™ [60% (w/v) iodixanol; Sigma Aldrich, St. Louis, MO] with 0.25 M sucrose/10 mM Tris, pH 7.5 to generate 40%, 20%, 10% and 5% (w/v) iodixanol solutions. The discontinuous iodixanol gradient was carefully arranged by sequentially layering 1 mL each of 40, 20 and 10% (w/v) iodixanol solutions, followed by 0.8 mL of the 5% iodixanol solution in 5PA centrifuge tubes (HITACHI). A 160 µL volume of crude-SaEVs suspension was overlaid on the discontinuous iodixanol gradient and ultracentrifuged for 16 h at 100,000×*g*, 4°C using a P55ST2 swing-out rotor. Six fractions (670 μL each) were collected from the top of the gradient. The substances in each fraction were collected and washed with PBS by ultracentrifugation at 100,000×*g*, 4°C for 2 h with a P55AT fixed angle rotor. The pellet was resuspended in an appropriate volume of ice-cold PBS. The SaEV-containing fraction was analyzed by electron microscopy. Negative staining was performed after applying the SaEVs on the copper grids with carbon-coated formvar supporting membrane. Then, the SaEVs were observed under an electron microscope (JEM-1230, JEOL, Tokyo, Japan). Particle concentration and size of SaEVs were analyzed using qNano instrument (Izon Science, Oxford, United Kingdom) according to the standard operating procedures. The purified SaEVs were diluted in Measurement Electrolytes (qNano reagent kit) containing 0.03% Tween 20 and analyzed using NP150 nanopore membranes in comparison with 200-nm calibration particles by running at 0.8 V. Data were analyzed using Izon Control Suite software version 3.3.2.2001.

### Protein detection

Proteins in the SaEVs were analyzed by SDS-PAGE, and protein concentration in the SaEVs was quantified using Bradford protein assay (Bio-Rad, Hercules, CA). Western blotting was performed to determine whether the superantigens [toxic shock syndrome toxin 1 (TSST-1) and staphylococcal enterotoxin C (SEC)] are present in the SaEVs. Both toxin-detecting antibodies have been produced in our laboratory from rabbits immunized with each toxin [14–17]. Horseradish peroxidase-conjugated anti-rabbit IgG (MP Biomedicals, MP Biomedicals, Irvine, CA) was used as secondary antibody.

### Proteomic analysis of SaEVs

Proteomic analyses of SaEVs (16 μg) were performed in triplicate by liquid chromatography-tandem mass spectrometry using a nanoLC Eksigent 400 system coupled to a TripleTOF6600 mass spectrometer (AB Sciex, Framingham, MA). Briefly, the acetone precipitated proteins of SaEVs were denatured with 50% trifluoroethanol and reduced using 4 mM dithiothreitol. Free cysteine residues were alkylated before trypsinization, and the desalted peptides were separated using liquid chromatography. The mass spectrometer was operated in information-dependent acquisition mode. Acquired spectra were searched against the *S. aureus* 834 proteins (NCBI Genbank: AP024170) using the Paragon algorithm embedded in the ProteinPilot 5.0.1 software program (AB Sciex). Positive identiﬁcations were considered when identiﬁed proteins and peptides reached a 1% local false discovery rate. Molecular weight and subcellular localization of each identified protein was predicted by Compute pI/Mw tool-ExPASy (https://web.expasy.org/compute_pi/), PsortB v.3.0 (https://www.psort.org/psortb/), respectively.

### Spleen cell preparation and cell culture

Spleen was collected and squeezed in RPMI 1640 medium (Nissui Pharmaceutical Co., Tokyo, Japan). Spleen cells were filtered through stainless steel mesh (size, 100 μm). Then, the erythrocytes were lysed with 0.85% NH_4_Cl. After washing 3 times with RPMI 1640 medium, the spleen cells were suspended in RPMI 1640 medium supplemented with 10% fetal calf serum (FCS; JRH Biosciences, Lenexa, KS), 0.03% L-glutamine (Wako Pure Chemical Industries, Osaka, Japan), 1×Antibiotic-Antimycotic (Gibco; Thermo Fisher, Waltham, MA), and were maintained in 5% CO_2_ incubator at 37°C. RAW264.7 cells, a mouse macrophage cell line, were cultured at 37°C under 5% CO_2_ in Dulbecco’s modified Eagle medium (DMEM; Nissui Pharmaceutical Co.), supplemented with 10% FCS, 0.03% of L-glutamine and 1 × Antibiotic-Antimycotic. For cytokine assays, the spleen cells and RAW264.7 cells were seeded at 2 × 10^6^ cells/well in a 24-well culture plate and incubated at 37°C, 5% CO_2_ with 0–5 μg/mL SaEVs for 72 h. The culture supernatants were harvested and stored at −80°C until the cytokine assays were performed.

### RNA interference of TLRs

RAW267.4 cells in the above mentioned DMEM were seeded in 24-well plate (2 × 10^5^ cells/100 μL/well), and then incubated under normal growth conditions (37°C, 5%CO_2_) shortly before transfection. Gene silencing was carried out using HiPerFect Transfection Reagent (QIAGEN GmbH, Hilden, Germany) according to the manufacturer’s instructions. Briefly, small interfering RNA (siRNA) for each TLR (QIAGEN GmbH) and negative control siRNA that has no homology to any known mammalian gene were diluted to be 375 ng/100 μL in DMEM without FCS. Six μL of HiPerFect Transfection Reagent was added into the diluted siRNA and mixed vigorously. After incubation for 5 min at room temperature, 100 μL of the transfection complexes was dropped onto the cells. The cells were continuously incubated for 6 h at 37°C under 5%CO_2_. Afterward, 400 μL of DMEM containing 10% FCS was added to the cells. The reduced gene expression was confirmed by real-time quantitative reverse transcription PCR (RT-qPCR). The mRNA level of each TLR was evaluated using glyceraldehyde-3-phosphate dehydrogenase as a reference gene. At 48 h of RNA interference, the TLR-silenced cells were incubated with 0 and 2 μg SaEVs for 72 h. Then, the culture supernatants were harvested and stored at −80°C. The cytokine assays were performed to examine the impairment of cytokine production.

### Cytotoxic effect of SaEVs

RAW264.7 cells (5 × 10^4^ cells/100 μL/well) were seeded in 96-well plate and incubated with 0, 0.04, 0.2, 1 and 5 μg/mL SaEVs at 37°C, 5%CO_2_. After 24 h of incubation, cell viability was determined using WST-1 Cell Proliferation Reagent (Sigma-Aldrich) according to the manufacturer’s instruction. Briefly, 10 μL of WST-1 reagent was added directly into each well and incubated at 37°C, 5%CO_2_ for 0.5–2 h. After colour development, the absorbance was measured at 450 nm using a microplate reader (Model 680, Bio-Rad, Richmond, CA). The absorbance values were subtracted with the background control (medium without RAW264.7 cells containing 0, 0.04, 0.2, 1 and 5 μg/mL SaEVs, respectively). Cell viability of 100% was referred to the cells with 0 μg/mL SaEVs.

### Cytokine assays

The titre of TNF-α, IL-6, IFN-γ and IL-17A was measured using Mouse ELISA kits purchased from Invitrogen, Carlsbad, CA. The protocol followed the manufacturer’s instructions. In order to investigate whether the cytotoxicity of SaEVs is associated with IL-1β, the production of this cytokine was determined by Mouse IL-1β ELISA kit (Invitrogen). For the Th2-related cytokines, IL-4 and IL-5 were determined using IL-4 Mouse ELISA kit (Invitrogen) and IL-5 Mouse ELISA kit (Invitrogen), respectively by following the manufacturer’s instructions.

### Immunization of mice with SaEVs and *S. aureus* infection

The purified SaEVs were diluted in PBS and emulsified with Alum adjuvant (Pierce, Rockford, IL) at a ratio of 1:1. Six-week-old mice were subcutaneously administrated with 200 μL of the emulsion containing 10 μg SaEVs or PBS (as a control) on Days 0 and 14. On Day 10 after the second immunization, mice were intravenously infected with 1 × 10^8^ CFU of MRSA 834 (200 μL of 5 × 10^8^ CFU/mL) and their survival was monitored for 8 d. At 3 h after infection, the proinflammatory cytokines in serum and spleen homogenate were determined by ELISA. To investigate whether Th2-related immune response is triggered and IgE is produced in the SaEV-immunized mice, spleen and serum were collected on Day 5 after the second immunization. The serum was kept at −80°C until IgE determination. The spleen cells were prepared as mentioned above, and stimulated with 0, 0.1 and 1.0 μg/mL SaEVs *ex vivo* for 72 h. Then, production of IL-4, IL-5 and IL-6 in the cell supernatant was determined by ELISA.

### Determination of total IgE

To investigate whether SaEV-immunization stimulates IgE production, total IgE titre in the serum was determined using ELISA MAX™ Standard Set Mouse IgE (BioLegend, San Diego, CA) according to manufacturer instruction. Pre-existing baseline of IgE in mouse sera was from naïve mice, which were randomly selected from the same lot of experimental mice without bias and maintained under the same conditions as the experimental mice.

### Statistical analysis

Statistical tests undertaken for individual experiments are mentioned in the figure legends. A *P*-value of less than 0.05 was considered statistically significant.

## Results

### Purification of SaEVs

**Figure 1.**
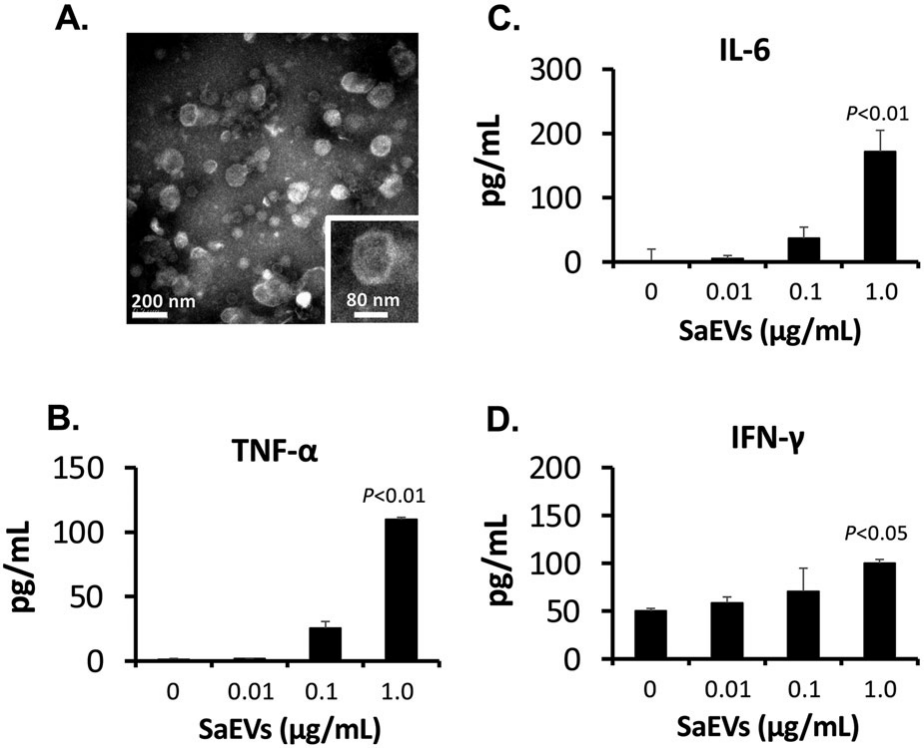
Stimulation of proinflammatory cytokines from naïve mouse spleen cells by SaEVs. (A) Negative staining transmission electron micrograph of purified SaEVs. The SaEVs were collected from culture supernatant of MRSA 834 and separated from non-membranous proteins, protein aggregates, and denatured membrane vesicles by step-gradient ultracentrifugation using OptiPrep™ Density Medium. (B–C) Production of proinflammatory cytokines from naïve mouse spleen cells after stimulated with SaEVs. Spleen cells from naïve BALB/c mice were prepared and adjusted to 2 × 10^6^ cells/mL. They were incubated with 0, 0.01, 0.1 and 1 μg/mL SaEVs for 72 h. The production of (B) TNF-α, (C) IL-6 and (D) IFN-γ in the culture supernatants was determined by ELISA (*n* = 6 from 2-independent experiments). *P*-value was calculated using ANOVA and *post hoc* with Dunnett’s test.

Crude particles from the culture supernatant of MRSA 834 were harvested by ultracentrifugation. Thereafter, SaEVs were separated from the denatured membrane vesicles, protein aggregates and non-membranous proteins by step-gradient ultracentrifugation using OptiPrep™ Density Medium. Six fractions (F1 to F6 from the top of the gradient) were collected, washed and resuspended in an appropriate volume of PBS based on pellet size (80–800 μL; Supplementary Table S1). Most of proteins were found in F3, F4 and F5 as shown by protein concentration (Supplementary Table S1) and the protein pattern from silver-stained SDS-PAGE (Supplementary Figure S1). Negative-staining transmission electron microscopy (Supplementary Figure S2) demonstrates that most SaEVs were separated in F5. Small particles were found in F3 and F4, whereas large membrane fragments were found in F6. From 4.8 L of the culture supernatant, approximately 324 μg total protein in F5 (SaEV-containing fraction) was obtained (Supplementary Table S1). No bacterial contamination was confirmed after inoculating a portion of the purified SaEVs (F5) into tryptic soy broth and culturing at 37°C for 48 h. Electron micrograph of the purified SaEVs (F5) in Figure 1(A) reveals that more than 95% were enclosed vesicles. The diameters of SaEVs are in a range of 78–159 nm with an average size at 101 ± 11.8 nm (Supplementary Figure S3). The particle concentration of SaEVs is approximately 5.6 × 10^7^ particles/1 μg protein.

### SaEVs stimulate host immune response via TLRs 2, 4 and 9

To investigate the effect of SaEVs on host immune response, spleen cells from naïve mice were prepared and treated with purified SaEVs. At 72 h of incubation, the production of proinflammatory cytokines in the culture supernatant was determined by ELISA. As shown in Figure 1(B–D), SaEVs stimulated the production of TNF-α, IL-6 and IFN-γ from naïve mouse spleen cells in a dose-dependent manner. Among all virulence factors of *S. aureus*, superantigens are considered to be major factors that induce proinflammatory cytokine production [1]. TSST-1 and SEC are two major superantigens that have been detected in the MRSA 834 strain [14–17]. Therefore, we further investigated whether the TSST-1 and SEC are present in the purified SaEVs. Western blot analysis in Supplementary Figure S4 shows that almost no positive signal of TSST-1 and SEC was detected in the purified SaEVs, even though these toxins were detected in crude particles from the culture supernatant of MRSA 834. The results indicate that these toxins were not encapsulated into SaEVs, and they were separated during Optiprep cleaned process.

**Figure 2.**
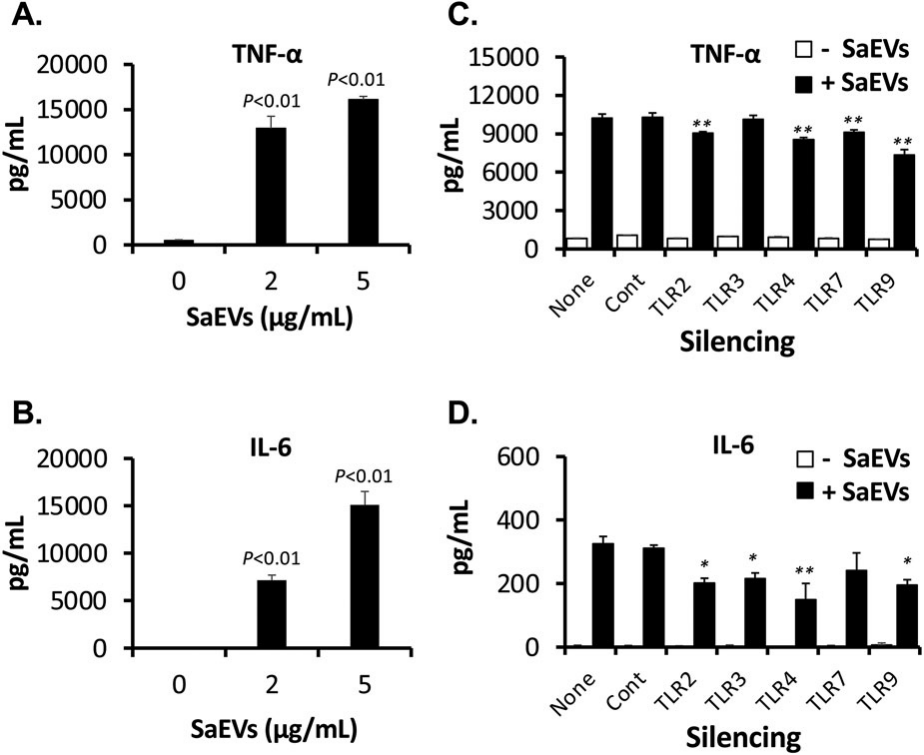
Stimulation of proinflammatory cytokines from mouse macrophages, RAW264.7 cells, by SaEVs via TLRs 2, 4 and 9. (A–B) RAW264.7 cells were prepared and adjusted to 2 × 10^6^ cells/mL. They were incubated with 0, 2 and 5 μg/mL SaEVs for 72 h. The production of (A) TNF-α and (B) IL-6 in the culture supernatants was determined by ELISA (*n* = 6 from 2-independent experiments). (C–D) Gene silencing of each indicated TLR was carried out using HiPerFect Transfection Reagent (QIAGEN GmbH, Hilden, Germany) according to the manufacturer’s instructions. At 48 h of silencing, the TLR-silenced cells were incubated with 0 and 2 μg/mL SaEVs for 72 h and the production of (C) TNF-α and (D) IL-6 in the culture supernatants was determined by ELISA (*n* = 6 from 2-independent experiments). None: non-transfection; Cont: negative control siRNA. *P* value was calculated using ANOVA and *post hoc* with Dunnett’s test. *: *P* < 0.05, **: *P* < 0.01.

In addition to the spleen cells, SaEVs remarkably stimulated mouse macrophages, RAW264.7 cells, to produce TNF-α and IL-6 (Figure 2(A–B)). It should be noted that, IFN-γ production could not be detected in these SaEV-stimulated RAW264.7 cells (data not shown). In order to predict whether the peptidoglycans, lipoproteins, RNA or DNA within SaEVs are proinflammatory stimulating ligands, silencing experiment of the TLR2, TLR3, TLR4, TLR7, and TLR9 gene in RAW264.7 cells was performed. The reduced gene expression of each TLR at 48 h of transfection was confirmed (Supplementary Figure S5). Afterward, the TLR-silenced cells were treated with SaEVs for 72 h. Significant impairment of both TNF-α and IL-6 production in the culture supernatant of TLR-silenced cells suggests that SaEVs triggered the inflammatory signalling in mouse macrophages via TLRs 2, 4 and 9 (Figure 2(C–D)).

### Identification of proteins in SaEVs

A total of 225 proteins were identified in 16 μg purified SaEVs (Supplementary Table S2). Bioinformatic analyses revealed that 67.6% of SaEV proteins were cytoplasmic (*n* = 152), 16.4% were membrane proteins (*n* = 37), 10.2% has unknown localization (*n* = 23), 4.9% were extracellular proteins (*n* = 11), and 0.8% were cell wall associated proteins (*n* = 2). Several virulence factors were detected including gamma haemolysin (*hlgA*, *hlgC*), leukocidin LukGH (*lukG*, *lukH*), cytolysin (SA834_10390), phenol-soluble modulins (SA834_10510, *hld*), staphylocoagulase (*coa*), fibrinogen-binding protein (SA834_10340) and immunoglobulin-binding protein Sbi (*sbi*). Drug resistance- and other virulence-related proteins included PBP2a family beta-lactam-resistant peptidoglycan transpeptidase MecA (*mecA_1*), transcriptional regulator SarR (*sarR*), autolysin (SA834_09310) and lipoprotein (SA834_03030). It should be noted that the presence of superantigens including TSST-1 and SEC in SaEVs could not be detected by proteomic analysis.

### SaEVs induce host cell death

**Figure 3.**
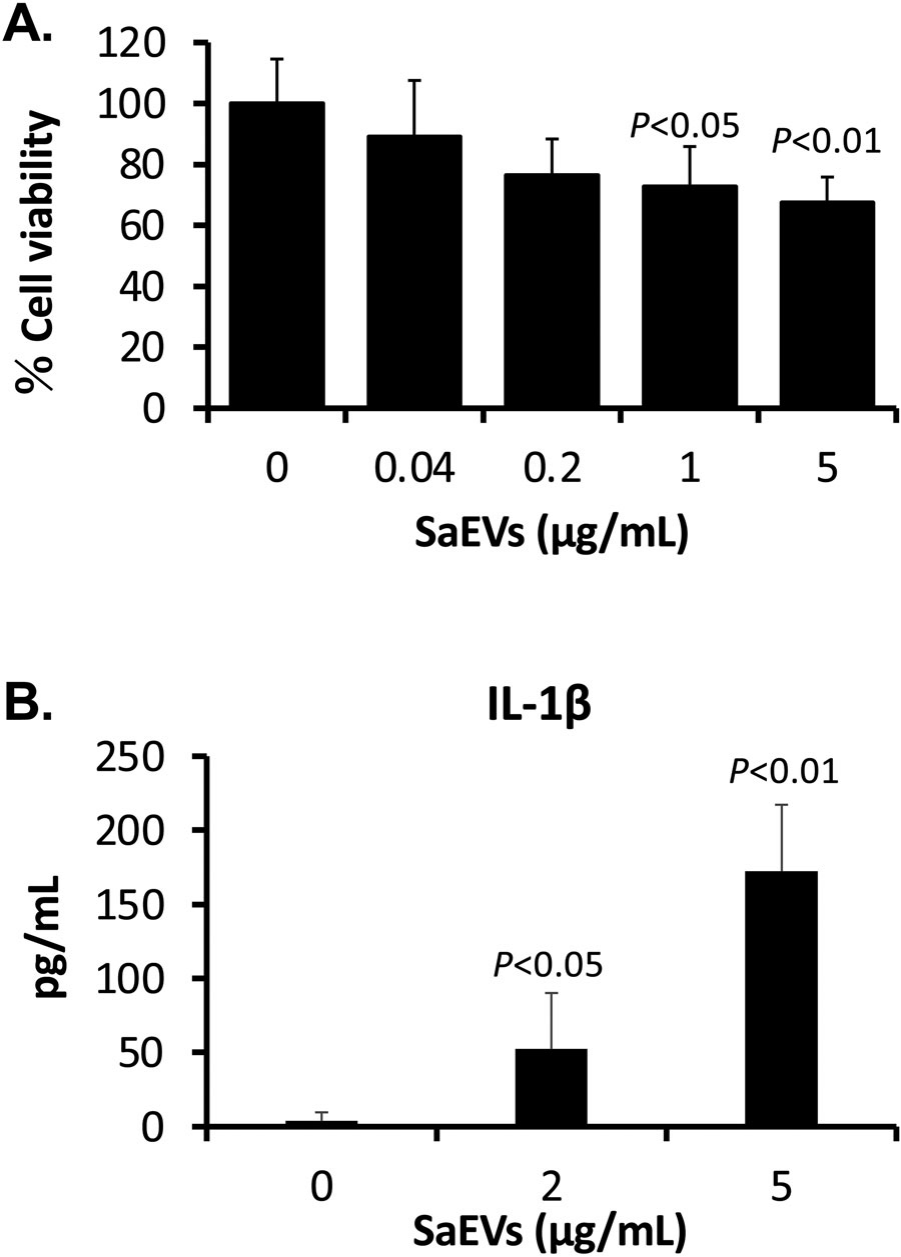
Cytotoxic effect of SaEVs to mouse macrophages. (A) RAW264.7 cells (5 × 10^4^ cells/100 μL/well) were seeded in 96-well plate and incubated with 0, 0.04, 0.2, 1 and 5 μg/mL SaEVs at 37°C under 5%CO_2_. At 24 of incubation, 10 μL of WST-1 reagent was added into each well. After colour development, the absorbance was measured at 450 nm. Cell viability in the reactions without SaEVs was calculated as 100% (*n* = 6 from 2-independent experiments). (B) RAW264.7 cells were prepared and adjusted to 2 × 10^6^ cells/mL. They were incubated with 0, 2 and 5 μg/mL SaEVs for 72 h. The production of IL-1β in the culture supernatants was determined by ELISA (*n* = 6 from 2-independent experiments). *P* value was calculated using ANOVA and *post hoc* with Dunnett’s test.

Regarding the results of TLR activation in RAW264.7 cells, cytotoxic effect of SaEVs to these mouse macrophages was further investigated. Cell viability of RAW264.7 cells was determined after incubation with SaEVs for 24 h. The result in Figure 3(A) reveals that SaEVs affected cell viability of mouse macrophages in a dose-dependent manner. At 1 and 5 μg SaEVs, the viability of RAW264.7 cells remained 73% and 67%, respectively. Taken together with TLR activation and proinflammatory cytokine production, SaEVs are considered to induce inflammation-related cell death. To confirm the involvement of IL-1β, the production of this cytokine was examined. As shown in Figure 3(B), SaEVs stimulated the IL-1β production from mouse macrophages in a dose-dependent manner.

### Hyperinflammatory shock occurs in SaEV-immunized mice after MRSA infection

**Figure 4.**
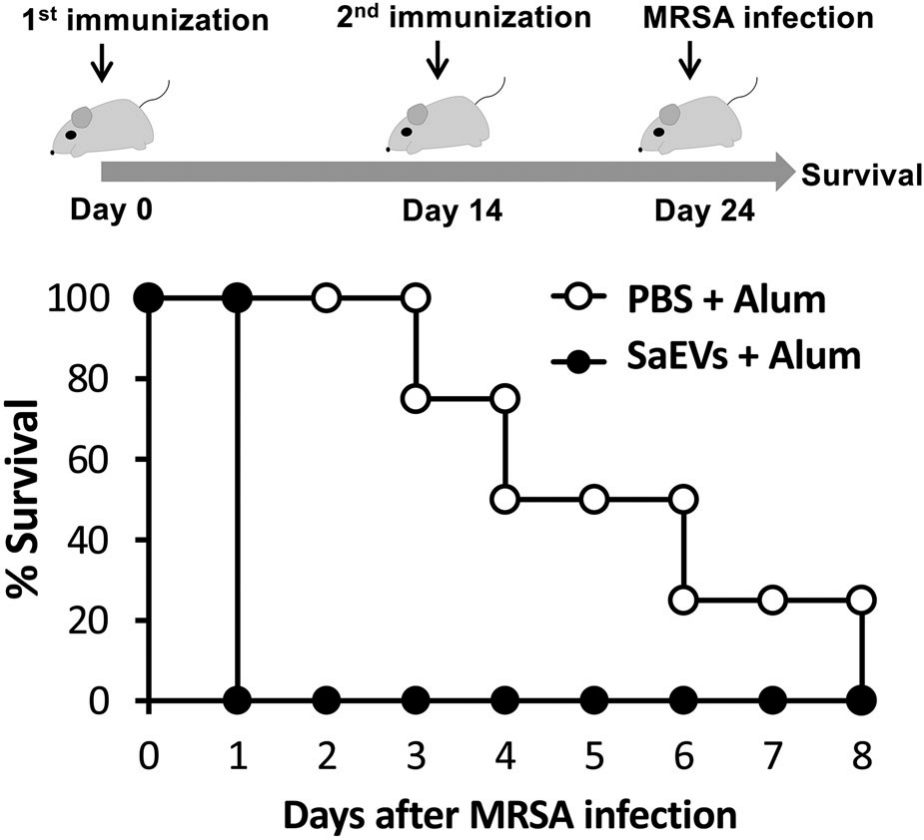
SaEV immunization fails to provide protective effect against MRSA infection. Mice were subcutaneously immunized with SaEVs and Alum twice on Day 0 and Day 14. Control mice were subcutaneously administrated with PBS and Alum. On Day 10 after the second immunization, 200 µL of MRSA (5 × 10^8^ CFU/mL) was intravenously infected, and survival of mice was monitored (*n* = 4–6 from 2-independent experiments).

**Figure 5.**
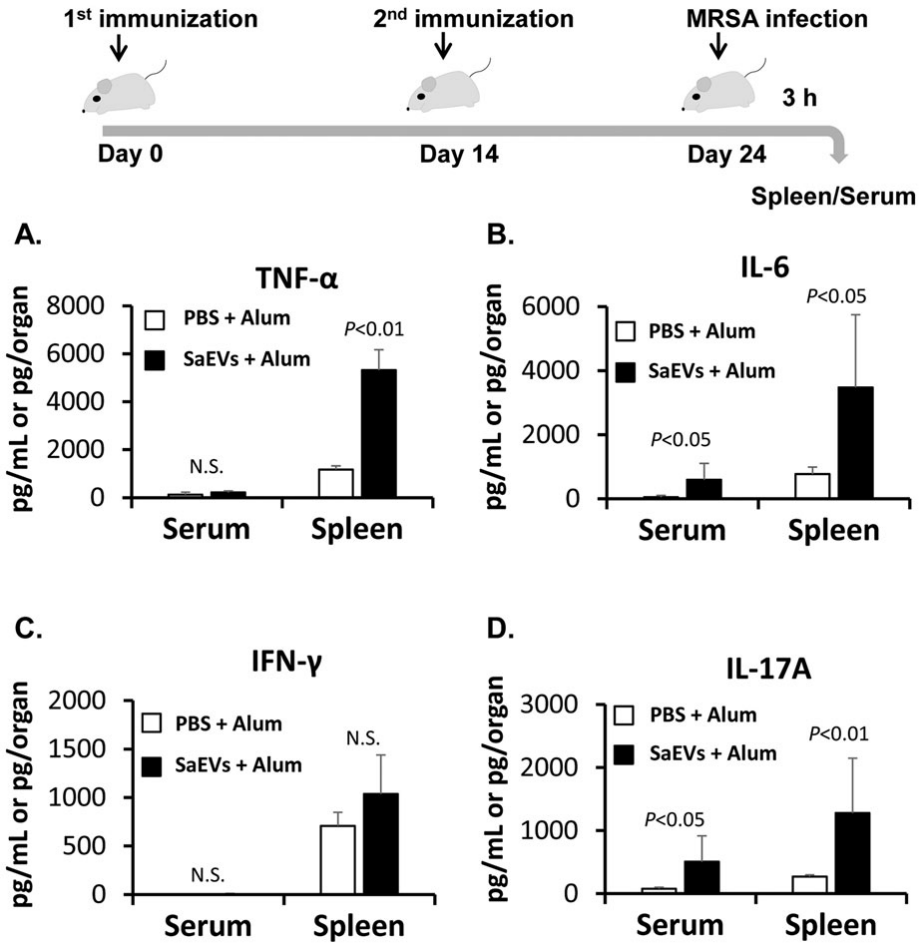
Inflammatory response in SaEV-immunized mice after MRSA infection. Mice were immunized with SaEVs followed by MRSA infection as described in Figure 4. At 3 h after infection, serum and spleen were collected. The titre of (A) TNF-α, (B) IL-6, (C) IFN-γ and (D) IL-17A was determined by ELISA (*n* = 4–6 from 2-independent experiments). *P*-value was calculated using student *t*-test.

Due to the biological effects of SaEVs, antibodies against SaEVs are thought to be effective in preventing and reducing the severity of MRSA infections. Therefore, mice were immunized with SaEVs in the presence of Alum adjuvant, and the protective effect against MRSA infection was examined. On Day 10 after the second immunization, mice were intravenously infected with lethal dose of MRSA, and the survival of mice was monitored. The results in Figure 4 show that all control mice (PBS + Alum) gradually died from Day 3 to Day 8 after MRSA infection. Surprisingly, all SaEV-immunized mice died on Day 1. These results indicate that SaEV immunization does not provide protective effect against MRSA infection. In contrast, hyperinflammatory shock seemed to be induced in these mice. Therefore, the inflammatory response in these mice was further investigated. At 3 h after MRSA infection, the titres of proinflammatory cytokines in the serum and spleen of SaEV-immunized mice were determined. As shown in Figure 5, the production of IL-6 and IL-17A in the serum and the production of TNF-α, IL-6 and IL-17A in the spleen of SaEV-immunized mice were significantly higher than that of control mice.

### Immunization of SaEVs stimulates IgE production and triggers Th2-related cytokine response

**Figure 6.**
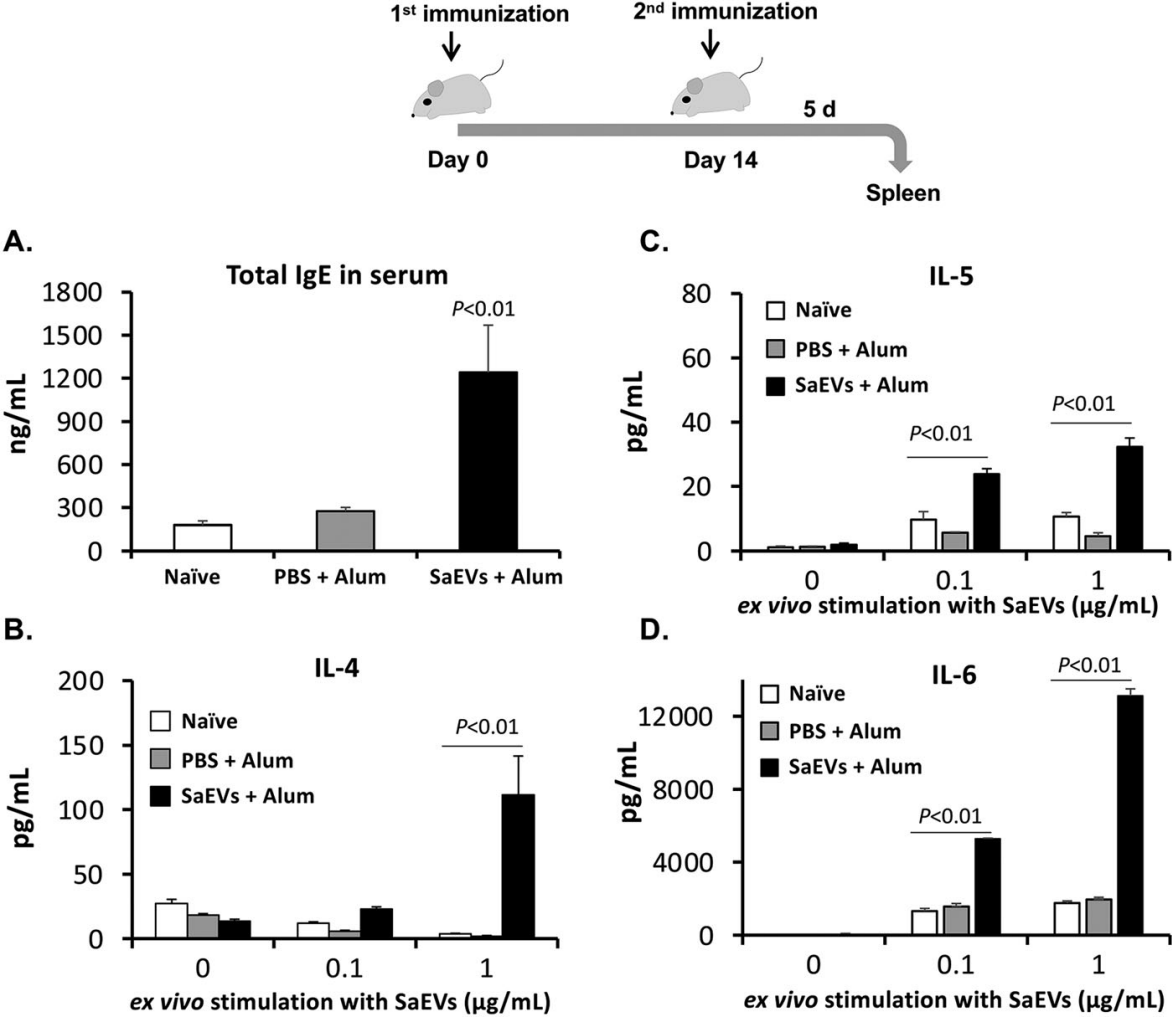
SaEV immunization stimulates IgE production and triggers Th2-related cytokine response. Mice were immunized with SaEVs as described in Figure 4. On Day 5 after the second immunization, serum and spleen were collected. (A) Total IgE in serum was determined by ELISA. (B–D) The spleen cells were prepared and adjusted to 2 × 10^6^ cells/mL. They were incubated with 0, 0.1 and 1.0 μg/mL SaEVs for 72 h. The production of (B) IL-4, (C) IL-5 and (D) IL-6 in the culture supernatants was determined by ELISA (*n* = 4–6 from 2-independent experiments). *P-*value was calculated using ANOVA and *post hoc* with Dunnett’s test.

Since SaEV-immunized mice developed inflammatory shock after MRSA infection, we further investigated IgE-mediated hypersensitivity after SaEV immunization. On Day 5 after the second immunization, the serum was collected for determination of IgE titre. Figure 6(A) demonstrates that the total IgE titre in the serum of SaEV-immunized mice was significantly higher than that of the naïve and non-SaEV (PBS + Alum) control mice. In addition to the serum, the spleen of SaEV-immunized mice was simultaneously collected. We found that the size of spleen from these mice was remarkably larger than that of the control mice (Supplementary Figure S6). After the preparation of spleen cells, *ex vivo* stimulation with SaEVs was performed. Due to the IgE titre in the serum, the production of Th2-related cytokines was focused. The results in Figure 6(B–D) reveal that *ex vivo* stimulation with 0.1 or 1 μg/mL SaEVs significantly induced the production of IL-4, IL-5 and IL-6 from the spleen cells isolated from SaEV-immunized mice. On the other hand, no production of these Th2-related cytokines was observed from the cells without *ex vivo* SaEV-stimulation (0 μg/mL SaEVs).

## Discussion

In the present study, SaEVs were purified from MRSA 834 for investigating the role on host immune response. Electron micrograph shows that the SaEVs fractionated in F5 of step-gradient ultracentrifugation had a high purity (Figure 1(A)). Although some contaminations may remain, most of large debris fragments were separated into F6 (Supplementary Figure S2). More than 95% of the particles in F5 are enclosed vesicles with the size of 30–160 nm. This size is comparable to the size of membrane vesicles from the previous report [7]. These purified SaEVs in F5 significantly stimulated the inflammatory response from mouse naïve spleen cells in a dose-dependent manner (Figure 1(B–D)). However, the small particles in the F3 and F4 at the same protein concentration also stimulated the comparable cytokine production (data not shown).

The superantigens including TSST-1 and SEC are key virulence factors of MRSA 834 that stimulate excessive proinflammatory cytokines such as IFN-γ and IL-2 via cross-linking between T cell receptor and MHC class II on antigen-presenting cells [18]. However, the stimulation of IFN-γ by SaEVs from mouse naïve spleen cells was not as high as that of TNF-α and IL-6 (Figure 1(B–D)). In addition, the presence of TSST-1 and SEC in the purified SaEVs could not be detected by proteomic analysis (Supplementary Table S2). In an absence of T cells, SaEVs also remarkably stimulated the IL-6 and TNF-α (Figure 2(A–B)) but not IFN-γ production from mouse macrophages, RAW264.7 cells. Furthermore, SaEVs isolated from a TSST-1-deficient mutant of MRSA 834 also stimulated the comparable TNF-α production to the SaEVs isolated from the wild type (Supplementary Figure S7). Taken together, it is possible that the molecules other than superantigens in the SaEVs of MRSA 834 stimulate the proinflammatory response.

TLRs are pattern recognition receptors that recognize bacterial and viral originated molecules, and mediate the initiation of innate and antigen-specific adaptive immune response [19]. To predict the ligands in SaEVs that stimulate immune response, gene expression of TLR2, TLR3, TLR4, TLR7 and TLR9 in RAW264.7 cells was silenced. Recognition of these selected TLRs covers most types of molecules present in EVs. TLR2 recognizes bacterial cell components such as peptidoglycans, lipoproteins and lipoteichoic acid [20]. TLR3 recognizes double-stranded RNA that is a viral infection-associated ligand and also double-stranded RNA from necrotic cells and intracellular bacteria [21]. Although lipopolysaccharide from Gram-negative bacteria is well known TLR4 ligand, some proteins from Gram-positive *Streptococcus pneumonia* have been shown to activate TLR4 [22,23]. TLR7 and TLR9 recognize single-stranded RNA and DNA, respectively [19]. The experiment of RNA interference demonstrates that TLRs 2, 4 and 9 are important for SaEV-mediated inflammatory response (Figure 2(C–D)). Thus, peptidoglycans, lipoproteins, bacterial proteins and/or single-stranded DNA in SaEVs are suggested to be the ligands that stimulate proinflammatory cytokine production from mouse macrophages. An impairment of TNF-α and IL-6 production in the TLR-silenced cells did not reach 50%. This might be due to the simultaneous activation of all three TLRs or the recovery of TLR expression during 72-h treatment of the SaEVs. TLR2 and TLR4 mainly locate on the cell surface, whereas TLR9 is in the endosome [19]. Therefore, it has possibility that the SaEVs may be initially disintegrated at around host cell surface. Then, the released peptidoglycans, lipoproteins and/or proteins from the SaEVs trigger the signalling of TLR2 and TLR4. On the other hand, single-stranded DNA in SaEVs may be released after endocytosis and stimulate TLR9 intracellularly. Wang et al. [11] have reported that EV-associated lipoproteins mediated TLR2 signalling to initiate NLRP3 inflammasome activation. Moreover, Schlatterer et al. [10] demonstrated that the surfactant-like molecules such as phenol-soluble modulins play a critical role to release lipoproteins from *S. aureus* via membrane vesicles, and the vesicle disruption at high detergent concentrations promotes the capacity of lipoproteins to activate TLR2. Although the release of lipoproteins or peptidoglycans from the SaEVs has not been proven in our study, the proteomic data in Supplementary Table S2 showed that SaEVs harbour lipoprotein (SA834_03030), autolysin (SA834_09310), proteases (*clpx*, *hslU*, SA834_16740, *hslV*) and phenol-soluble modulins (SA834_10510, *hld*). Recently, Bitto et al. [24] reported that DNA, RNA and peptidoglycan in the EVs from *S. aureus* NCTC 6571 are immunostimulators that activate the innate immune response. In that report, non-phagocytic epithelial cells (HEK-Blue cells) expressed with human TLRs containing an inducible NF-κB/AP1 secreted alkaline phosphatase reporter were used. This different detection system and also differences in EVs derived from different strains may lead to different results of our finding. As shown by Jeon et al. [9], EVs from different clinical *S. aureus* isolates not only carry several common proteins, but also carry strain-specific proteins, which may relate to different activity to the host cells. Further investigation of the signalling from TLRs as well as other cytosolic pattern recognition receptors such as NOD family proteins is needed to ascertain whether the other ligands in SaEVs from MRSA 834 promote inflammatory responses.

Wang et al. [11] reported that lipoproteins and pore-forming toxins including alpha toxin and leukocidins in EVs of *S. aureus* orchestrate to activate NLRP3 inflammasome via caspase 1, resulting in an induction of pyroptosis in human macrophage THP-1 cells. In our study, the activated forms of caspase 1 could not be detected because the apoptotic speck-like protein with a caspase-activating recruiting domain is deleted in the RAW264.7 cell line [25]. Thus, the relation of IL-1β for cytotoxicity of SaEVs in RAW 264.7 cells is still unclear. The release of IL-1β in RAW264.7 cells by SaEVs is probably involved in the other pathway such as caspase 11-mediated noncanonical inflammasome [26]. Due to the pathogenicity of SaEVs, these vesicles were considered as preventive target for severe MRSA infections. Unfortunately, immunization of SaEVs did not provide the protective effect against MRSA. In contrast, death of SaEV-immunized mice was found within 1 d after infection. At 3 h after MRSA infection, the titres of proinflammatory cytokines were significantly higher than that of control mice (Figure 5), suggesting an immediate induction of hyperinflammatory shock in the SaEV-immunized mice. These results indicate that SaEVs need to be detoxified before being used as a vaccine. Hyperinflammatory shock in the SaEV-immunized mice after MRSA infection (Figures 4 and 5) suggested the development of IgE-mediated systemic anaphylaxis, a severe life-threatening allergic reaction. To uncover this expectation, the total IgE in the serum and the production of Th2-related cytokines after *ex vivo* SaEV stimulation were confirmed (Figure 6). Taken together, our results demonstrate that SaEV immunization with Alum sensitizes allergic rather protective immunity. These results are unrelated to a previous report [27], which demonstrated that immunization of mice with *S. aureus*-derived membrane vesicles resulted in protective immunity in a mouse infection model. This may be due to the use of different *S. aureus* strain, which can lead to different types and amounts of vesicle-related antigens. Furthermore, mice were immunized in different ways. In the study of Askarian et al. [27], mice were immunized intraperitoneally without adjuvant, whereas in our study, mice were subcutaneously immunized with a mixture of SaEVs and Alum. It has been reported that Alum adjuvant not only enhances immunogenicity but also induces Th2 cell immune responses [28]. Our results suggest that the combination between SaEVs and Alum significantly triggers antigenic specific Th2 immunity in comparison with control (PBS + Alum) (Figure 6). By using naïve mouse spleen cells, SaEVs alone (without Alum adjuvant) stimulated IFN-γ production but the titre of this cytokine was lower than that of TNF-α and IL-6 (Figure 1(B–D)). In addition, dramatic TNF-α and IL-6 titres were released from mouse macrophages (Figure 2(A–B)). TNF-α is involved in the development of Th2 cells [29], whereas IL-6 promotes Th2 differentiation [30]. Therefore, these inflammatory cytokines from SaEV-stimulated innate immune cells are probably key factors for Th2 activation and subsequent allergy induction. *S. aureus* has been shown to be associated with chronic allergic diseases but the mechanism is unclear [31]. Our finding may link to new insights into the role of SaEVs associated with allergies in MRSA carriers. However, the human body under normal physiological conditions does not have aluminium adjuvants. At the same time, the persistence of *S. aureus* on human skin and in nasal cavity suggests that the human body is continuously exposed to a small amount of SaEVs. Therefore, it is imperative to conduct long-term experiments by appropriately administering SaEVs to mice without adjuvant in the future.

In conclusion, SaEVs derived from MRSA 834 act as an immunostimulant that induces inflammatory response. Subcutaneous immunization of mice with SaEVs and Alum adjuvant triggers Th2-related immune response and IgE-mediated hypersensitivity. Hyperinflammatory shock in these SaEV-immunized mice is developed after MRSA infection. Our study sheds new light on the role of SaEVs, which may associate with allergies in MRSA carriers.

## Supplementary Material

Supplementary_data.docx
