# Supplementary material for: Extracellular vesicles from methicillin resistant Staphylococcus aureus stimulate proinflammatory cytokine production and trigger IgE-mediated hypersensitivity
Source: Emerg Microbes Infect. 2021 Oct 8;10(1):2000–9. doi: 10.1080/22221751.2021.1991239 (PMC8547819; doi:10.1080/22221751.2021.1991239)
Supplement: Supplementary_data.docx [file TEMI_A_1991239_SM1680.docx]

**Table S1. Total protein in each fraction (F1 to F6) obtained after fractionation by step-gradient ultracentrifugation from 4.8 L of MRSA culture supernatant.**

| **Fraction** | **Protein concentration (mg/mL)** | **Suspended volume (μL)** | **Total protein**  **(μg)** |
| --- | --- | --- | --- |
| F1 | 0.001 | 80 | 0.11 |
| F2 | 0.022 | 80 | 1.78 |
| F3 | 0.417 | 200 | 83.52 |
| F4 | 0.487 | 300 | 146.17 |
| F5 | 0.405 | 800 | 324.08 |
| F6 | 0.047 | 100 | 4.73 |


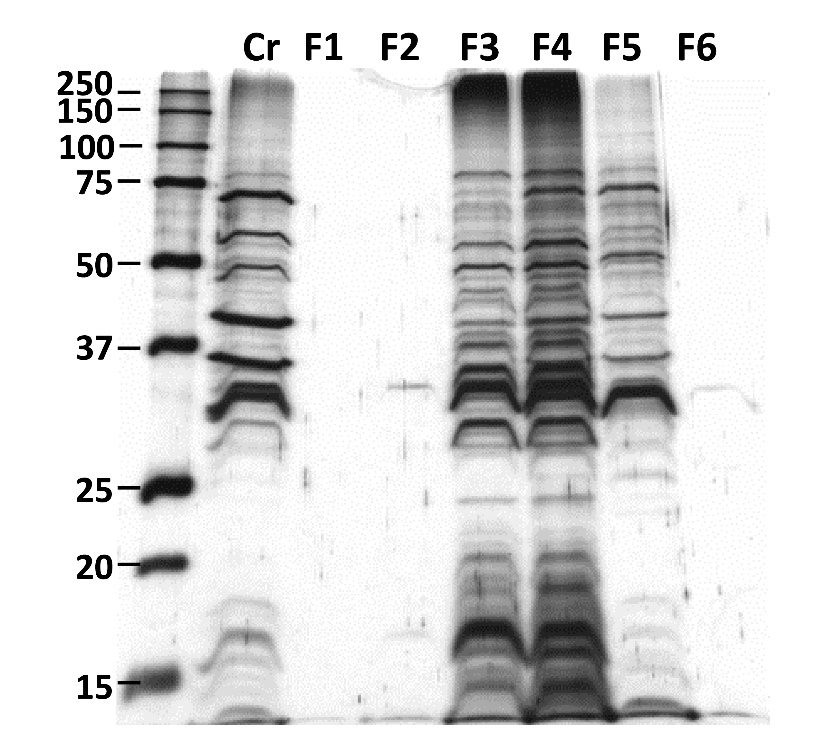


**Figure S1. Silver-stained SDS-PAGE of proteins from fractions 1 to 6 (F1 to F6, respectively) after fractionation by step-gradient ultracentrifugation.** One μg proteins from crude particles (Cr) and 5 μl of F1 to F6 were applied to 10% SDS-PAGE. After electrophoresis, the proteins were stained using Silver Stain Kit II (FUJIFILM Wako Chemicals). Molecular weight standards are indicated on the left (kDa).


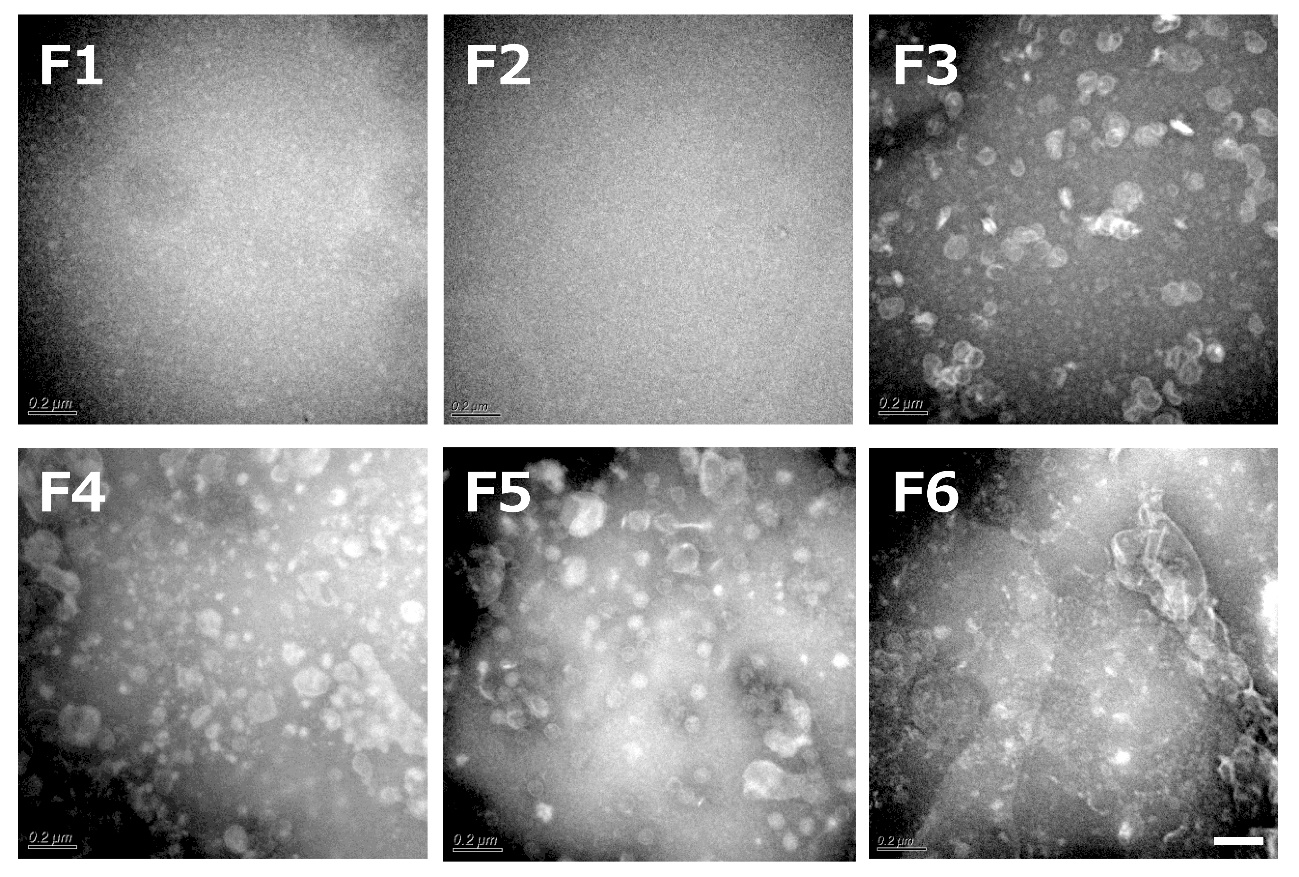


**Figure S2. Detection of SaEVs in fractions 1 to 6 (F1 to F6) after fractionation by step-gradient ultracentrifugation.** Majority of SaEVs were detected in F5 as shown by electron microscopy. Negative staining transmission electron microcopy was performed, and membrane-bound vesicles were observed under electron microscope. Scale bar = 200 nm.


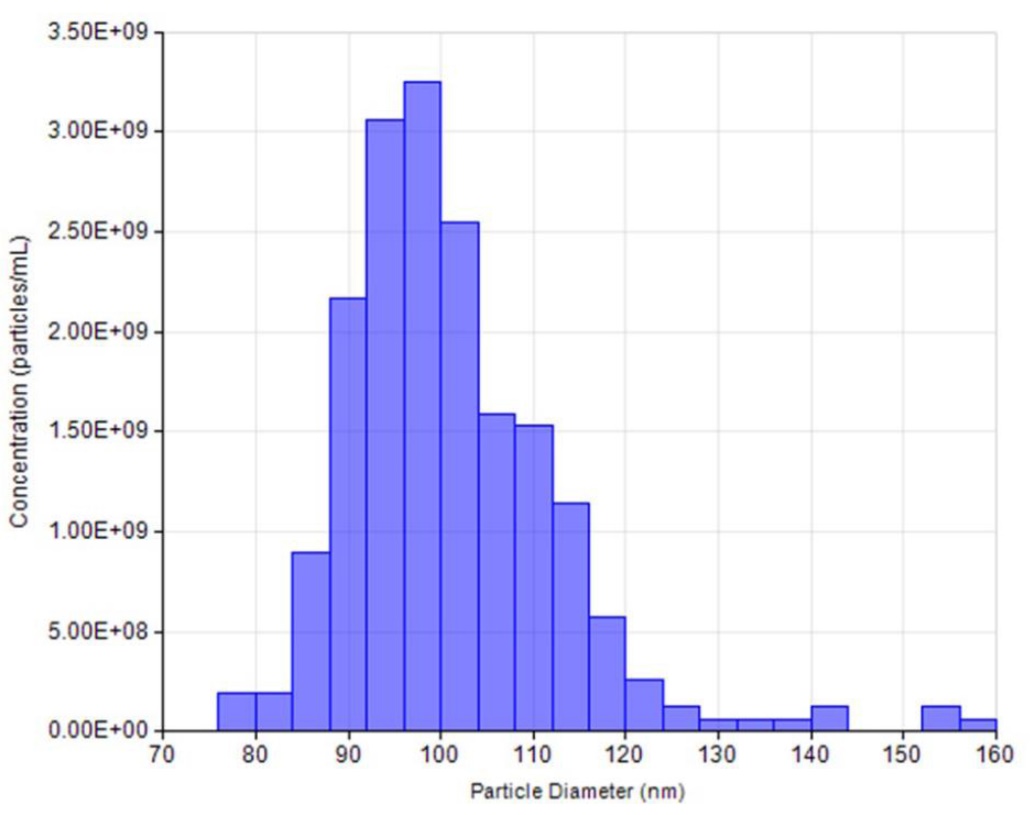


**Figure S3. Particle diameters of SaEVs in fraction 5 after analysis using qNano instrument (Izon Science, Oxford, United Kingdom).** The diameters of SaEVs are in a range of 78-159 nm with an average size at 101±11.8 nm.


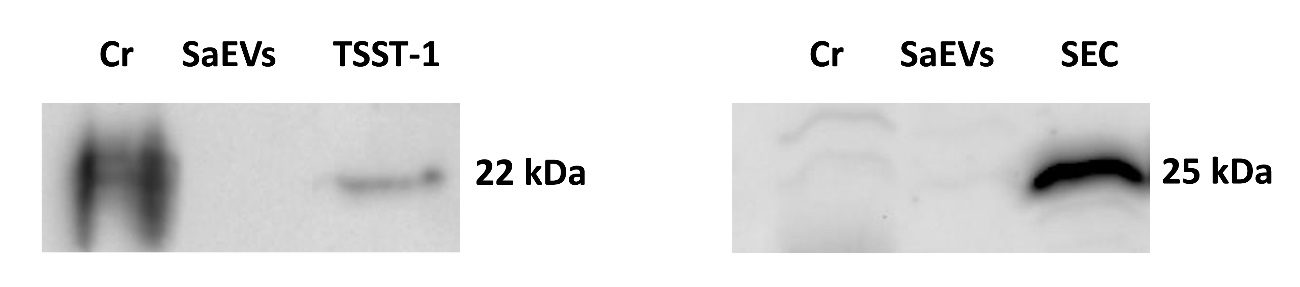


**Figure S4. Detection of superantigens in SaEVs by Western blotting.** Proteins from crude particles (Cr) and purified SaEVs (0.4 μg) were applied to SDS-PAGE. After transferring to the PVDF membrane, (A) toxic shock syndrome toxin 1 (TSST-1) and (B) staphylococcal enterotoxin C (SEC) were detected by anti-TSST-1 and anti-SEC antibody, respectively. Both antibodies were produced in our laboratory [10-13]. Recombinant TSST-1 (25 ng) and SEC (12.5 ng) were used as positive controls. Although both proteins were detected in the crude particles from MRSA 834 supernatant, almost no signal could be detected in SaEVs by Western blotting.


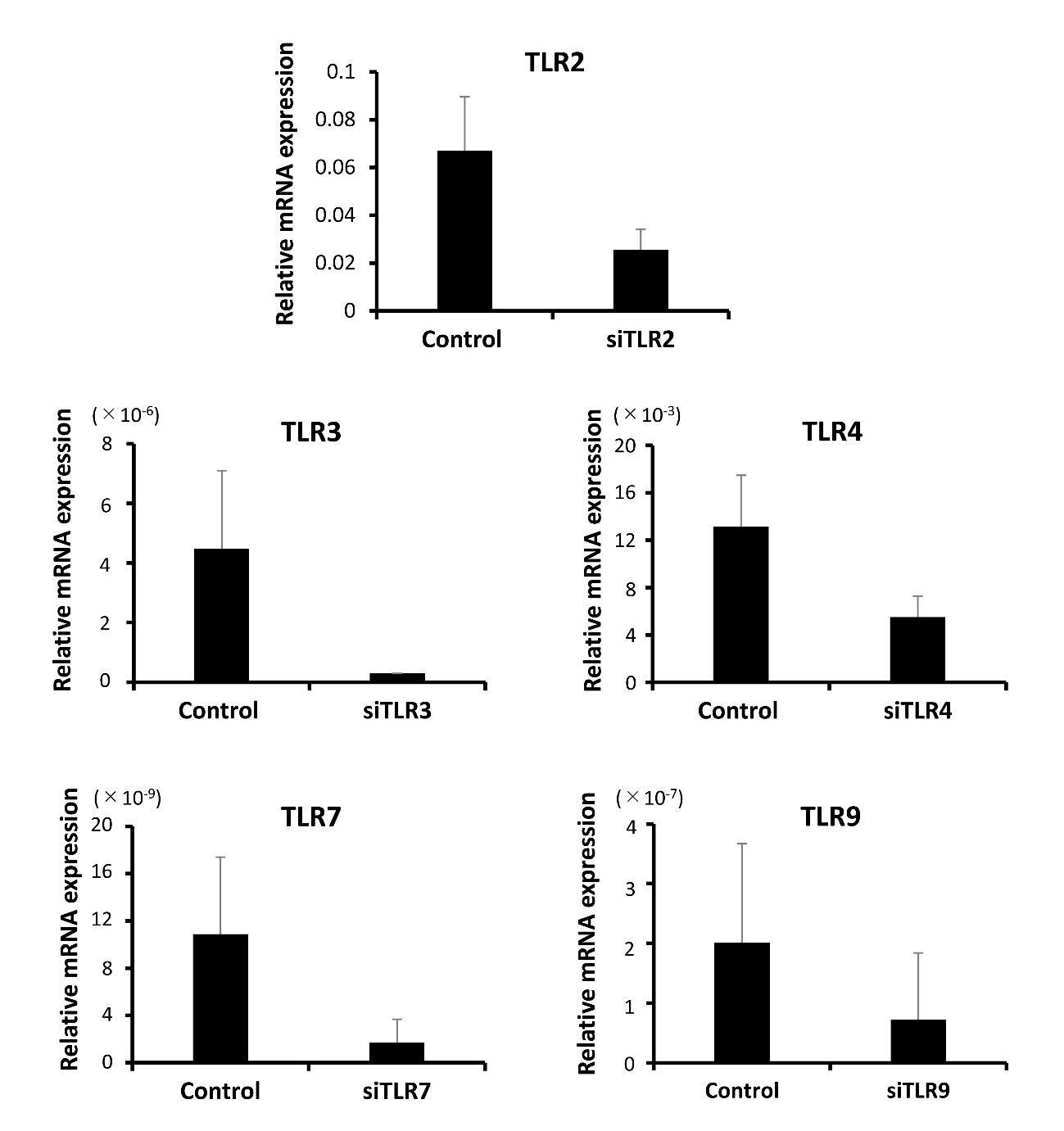
**Figure S5. Relative mRNA expression of TLRs after silencing with each specific siRNA.** Control is relative mRNA expression of each TLR gene after silencing with control siRNA (Qiagen). According to manufacturer’s instruction, RAW264.7 cells were prepared, and gene silencing was performed using HiPerFect Transfection (Qiagen). After 48 h, the effect of silencing was confirmed by real-time quantitative reverse transcription PCR. The mRNA expression level of each TLR was evaluated using glyceraldehyde-3-phosphate dehydrogenase as a reference gene.

**Table S2. Proteomic analysis of SaEVs.**

| **Protein** | **Gene name** | **MW (kDa)** | **Predicted localization** | **Peptides (95%)** | **%Cov**  **(95)** |
| --- | --- | --- | --- | --- | --- |
| DNA-directed RNA polymerase subunit beta' | *rpoC* | 135.41 | C | 58 | 52.11 |
| DNA-directed RNA polymerase subunit beta | *rpoB* | 133.22 | C | 37 | 35.84 |
| pyruvate dehydrogenase E1 component beta subunit | *phdB* | 35.25 | C | 26 | 70.46 |
| elongation factor Tu | *tuf* | 43.10 | C | 21 | 60.91 |
| dihydrolipoamide S-acetyltransferase component of pyruvate dehydrogenase complex E2 | *pdhC* | 46.37 | C | 21 | 39.77 |
| pyruvate kinase | *pyk* | 63.10 | C | 18 | 31.62 |
| pyruvate dehydrogenase E1 component alpha subunit | *pdhA* | 41.25 | C | 18 | 43.51 |
| type I glyceraldehyde-3-phosphate dehydrogenase | *gap_1* | 36.28 | C | 17 | 52.98 |
| elongation factor G | *fusA* | 76.61 | C | 14 | 29.00 |
| 30S ribosomal protein S2 | *rpsB* | 29.09 | C | 14 | 52.94 |
| alcohol dehydrogenase AdhP | *adhP* | 36.06 | C | 12 | 38.10 |
| F0F1 ATP synthase subunit alpha | *atpA* | 54.58 | C | 12 | 24.30 |
| phosphoglycerate kinase | *pgk* | 42.60 | C | 11 | 37.37 |
| threonine--tRNA ligase | *thrS* | 74.39 | C | 11 | 22.17 |
| Fe-S cluster assembly ATPase SufC | *sufC* | 28.28 | C | 11 | 52.17 |
| DEAD/DEAH box helicase | SA834_20280 | 56.94 | C | 11 | 25.49 |
| 50S ribosomal protein L5 | *rplE* | 20.27 | C | 10 | 65.36 |
| cysteine synthase A | *cysK* | 32.98 | C | 10 | 53.23 |
| uroporphyrinogen decarboxylase | *hemE* | 39.25 | C | 10 | 37.79 |
| Asp-tRNA(Asn)/Glu-tRNA(Gln) amidotransferase subunit GatB | *gatB* | 53.66 | C | 9 | 22.11 |

**Table S2. Proteomic analysis of SaEVs (continued).**

| **Protein** | **Gene name** | **MW (kDa)** | **Predicted localization** | **Peptides (95%)** | **%Cov**  **(95)** |
| --- | --- | --- | --- | --- | --- |
| lysine--tRNA ligase | *lysS* | 56.72 | C | 9 | 23.03 |
| pyridoxal 5'-phosphate synthase lyase subunit PdxS | *pdxS* | 31.99 | C | 8 | 34.58 |
| glutamine--fructose-6-phosphate transaminase (isomerizing) | *glmS* | 65.84 | C | 8 | 17.30 |
| H-type ferritin FtnA | *ftnA* | 19.59 | C | 8 | 28.31 |
| serine hydroxymethyltransferase | *glyA* | 45.17 | C | 7 | 19.90 |
| Asp-tRNA(Asn)/Glu-tRNA(Gln) amidotransferase subunit GatA | *gatA* | 52.80 | C | 7 | 18.35 |
| D-alanyl-lipoteichoic acid biosynthesis protein DltD | *dltD* | 44.93 | C | 7 | 24.04 |
| formate C-acetyltransferase | *pflB* | 84.86 | C | 7 | 11.21 |
| DNA gyrase subunit B | *gyrB* | 72.44 | C | 7 | 10.73 |
| formate--tetrahydrofolate ligase | SA834_16780 | 59.87 | C | 7 | 14.77 |
| ribonuclease J | SA834_09670 | 62.67 | C | 7 | 20.18 |
| acetyl-CoA carboxylase biotin carboxylase subunit | *accC* | 50.05 | C | 6 | 18.85 |
| bifunctional threonine ammonia-lyase/L-serine ammonia-lyase TdcB | *tdcB* | 37.15 | C | 6 | 22.54 |
| IMP dehydrogenase | *guaB* | 52.85 | C | 6 | 14.96 |
| acetate kinase | *ackA* | 44.06 | C | 6 | 19.75 |
| NAD-specific glutamate dehydrogenase | *gudB* | 45.76 | C | 6 | 17.87 |
| proline--tRNA ligase | *proS* | 63.86 | C | 6 | 11.82 |
| cell division protein FtsZ | *ftsZ* | 41.04 | C | 5 | 15.90 |
| CTP synthase | *pyrG* | 60.00 | C | 5 | 10.26 |
| L-lactate dehydrogenase | *lctE* | 34.57 | C | 5 | 18.61 |
| 50S ribosomal protein L1 | *rplA* | 24.71 | C | 5 | 33.48 |
| flotillin-like protein FloA | *floA* | 35.20 | C | 5 | 18.54 |

**Table S2. Proteomic analysis of SaEVs (continued).**

| **Protein** | **Gene name** | **MW (kDa)** | **Predicted localization** | **Peptides (95%)** | **%Cov**  **(95)** |
| --- | --- | --- | --- | --- | --- |
| 50S ribosomal protein L10 | *rplJ* | 17.71 | C | 5 | 36.75 |
| 30S ribosomal protein S13 | *rpsM* | 13.72 | C | 4 | 31.40 |
| aminomethyl-transferring glycine dehydrogenase subunit GcvPA | SA834_14850 | 49.71 | C | 4 | 10.04 |
| glycine--tRNA ligase | *glyS* | 53.62 | C | 4 | 8.86 |
| ATP-dependent Clp protease ATP-binding subunit ClpX | *clpX* | 46.30 | C | 4 | 11.67 |
| 50S ribosomal protein L2 | *rplB* | 30.16 | C | 4 | 27.44 |
| 30S ribosomal protein S7 | *rpsG* | 17.79 | C | 4 | 30.13 |
| tRNA guanosine(34) transglycosylase Tgt | *tgt* | 43.31 | C | 4 | 11.61 |
| type 2 isopentenyl-diphosphate Delta-isomerase | SA834_22890 | 38.87 | C | 4 | 9.74 |
| DUF948 domain-containing protein | SA834_16850 | 18.00 | C | 4 | 25.15 |
| 30S ribosomal protein S5 | *rpsE* | 17.74 | C | 4 | 36.14 |
| ATP-dependent protease ATPase subunit HslU | *hslU* | 52.33 | C | 4 | 10.49 |
| alanine dehydrogenase | *ald_1* | 40.23 | C | 4 | 13.44 |
| 50S ribosomal protein L6 | *rplF* | 19.79 | C | 4 | 29.21 |
| carbamoyl-phosphate synthase large subunit | *pyrAB* | 117.17 | C | 4 | 3.78 |
| uracil phosphoribosyltransferase | *upp* | 23.05 | C | 4 | 23.44 |
| molecular chaperone DnaK | *dnaK* | 66.36 | C | 4 | 10.16 |
| hypoxanthine phosphoribosyltransferase | *hpt* | 20.15 | C | 4 | 27.37 |
| DNA topoisomerase IV subunit B | *parE* | 74.54 | C | 3 | 4.51 |
| glycerol-3-phosphate dehydrogenase/oxidase | SA834_11790 | 62.37 | C | 3 | 5.75 |
| 50S ribosomal protein L22 | *rplV* | 12.83 | C | 3 | 16.24 |

**Table S2. Proteomic analysis of SaEVs (continued).**

| **Protein** | **Gene name** | **MW (kDa)** | **Predicted localization** | **Peptides (95%)** | **%Cov**  **(95)** |
| --- | --- | --- | --- | --- | --- |
| GTP-sensing pleiotropic transcriptional regulator CodY | *codY* | 28.76 | C | 3 | 9.34 |
| preprotein translocase subunit SecA | *secA* | 95.96 | C | 3 | 3.91 |
| 30S ribosomal protein S10 | *rpsJ* | 11.58 | C | 3 | 31.37 |
| 50S ribosomal protein L29 | *rpmC* | 8.09 | C | 3 | 50.72 |
| phosphopyruvate hydratase | *eno* | 47.12 | C | 3 | 8.30 |
| 30S ribosomal protein S9 | *rpsI* | 15.29 | C | 3 | 34.62 |
| universal stress protein | SA834_16560 | 18.47 | C | 3 | 24.10 |
| 1,4-dihydroxy-2-naphthoyl-CoA synthase | *menB* | 30.41 | C | 3 | 10.99 |
| bifunctional cystathionine gamma-lyase/homocysteine desulfhydrase | SA834_04300 | 41.25 | C | 3 | 11.32 |
| translation initiation factor IF-2 | *infB* | 77.86 | C | 3 | 3.97 |
| octopine dehydrogenase | SA834_22440 | 40.74 | C | 3 | 7.50 |
| asparagine--tRNA ligase | *asnS* | 49.13 | C | 3 | 7.21 |
| 50S ribosomal protein L14 | *rplN* | 13.14 | C | 3 | 18.85 |
| RNA polymerase sigma factor RpoD | *rpoD* | 42.17 | C | 3 | 13.59 |
| cell division protein FtsA | *ftsA* | 52.93 | C | 3 | 5.75 |
| phage portal protein | SA834_19100 | 44.50 | C | 3 | 11.37 |
| dihydrolipoamide dehydrogenase component of pyruvate dehydrogenase E3 | *pdhD* | 49.45 | C | 2 | 4.27 |
| signal transduction protein TraP | *traP* | 19.55 | C | 2 | 10.78 |
| 30S ribosomal protein S8 | *rpsH* | 14.83 | C | 2 | 14.39 |
| L-lactate dehydrogenase | SA834_25500 | 34.43 | C | 2 | 6.27 |
| glutamine-ammonia ligase | *glnA* | 50.85 | C | 2 | 4.26 |
| 30S ribosomal protein S3 | *rpsC* | 24.10 | C | 2 | 10.14 |

**Table S2. Proteomic analysis of SaEVs (continued).**

| **Protein** | **Gene name** | **MW (kDa)** | **Predicted localization** | **Peptides (95%)** | **%Cov**  **(95)** |
| --- | --- | --- | --- | --- | --- |
| pantetheine-phosphate adenylyltransferase | *coaD* | 18.37 | C | 2 | 11.87 |
| rRNA pseudouridine synthase | SA834_14410 | 27.97 | C | 2 | 9.39 |
| hypothetical protein | SA834_19410 | 17.31 | C | 2 | 13.51 |
| 30S ribosomal protein S1 | *rpsA* | 43.31 | C | 2 | 8.44 |
| 50S ribosomal protein L20 | *rplT* | 13.67 | C | 2 | 16.10 |
| F0F1 ATP synthase subunit epsilon | *atpC* | 14.84 | C | 2 | 22.39 |
| tyrosine--tRNA ligase | *tyrS* | 47.60 | C | 2 | 5.24 |
| 30S ribosomal protein S15 | *rpsO* | 10.61 | C | 2 | 39.33 |
| succinate dehydrogenase iron-sulfur subunit | *sdhB* | 30.58 | C | 2 | 7.75 |
| **HTH-type transcriptional regulator SarR** | *sarR* | 13.67 | C | 2 | 19.13 |
| cysteine desulfurase | SA834_07890 | 46.32 | C | 2 | 6.30 |
| ATP-dependent 6-phosphofructokinase | SA834_16450 | 34.84 | C | 2 | 6.21 |
| 50S ribosomal protein L16 | *rplP* | 16.24 | C | 2 | 19.44 |
| serine hydrolase family protein | SA834_17740 | 21.47 | C | 2 | 11.41 |
| D-lactate dehydrogenase | SA834_24710 | 36.71 | C | 2 | 5.45 |
| 2-oxo acid dehydrogenase subunit E2 | SA834_14650 | 46.79 | C | 2 | 7.55 |
| aminotransferase class I/II-fold pyridoxal phosphate-dependent enzyme | SA834_18810 | 48.12 | C | 2 | 6.78 |
| aspartate carbamoyltransferase | *pyrB* | 33.26 | C | 2 | 7.51 |
| thiamine pyrophosphate-dependent dehydrogenase E1 component subunit alpha | SA834_14670 | 36.20 | C | 2 | 4.85 |

**Table S2. Proteomic analysis of SaEVs (continued).**

| **Protein** | **Gene name** | **MW (kDa)** | **Predicted localization** | **Peptides (95%)** | **%Cov**  **(95)** |
| --- | --- | --- | --- | --- | --- |
| ACP S-malonyltransferase | *fabD* | 33.65 | C | 2 | 11.36 |
| aminoglycoside O-nucleotidyltransferase ANT(4')-Ia | SA834_00320 | 28.17 | C | 2 | 9.72 |
| 2,3-bisphosphoglycerate-independent phosphoglycerate mutase | *pgm* | 56.45 | C | 2 | 3.96 |
| 30S ribosomal protein S11 | *rpsK* | 13.88 | C | 2 | 10.08 |
| DNA-binding protein HU | *hu* | 9.63 | C | 2 | 34.44 |
| glycine dehydrogenase subunit 2 | SA834_14840 | 54.78 | C | 2 | 7.35 |
| glutamate-1-semialdehyde 2,1-aminomutase | *hemL* | 46.39 | C | 1 | 2.57 |
| translation initiation factor IF-3 | *infC* | 20.21 | C | 1 | 7.43 |
| bleomycin binding protein | SA834_00310 | 15.21 | C | 1 | 8.96 |
| glycine C-acetyltransferase | SA834_05200 | 42.89 | C | 1 | 3.29 |
| redox-regulated ATPase YchF | *ychF* | 40.59 | C | 1 | 2.74 |
| adenine phosphoribosyltransferase | *apt* | 19.12 | C | 1 | 7.56 |
| hypothetical protein | SA834_15210 | 27.23 | C | 1 | 4.31 |
| aldehyde dehydrogenase family protein | SA834_20690 | 51.97 | C | 1 | 2.32 |
| 23S rRNA (adenine(2503)-C(2))-methyltransferase RlmN | *rlmN* | 41.90 | C | 1 | 3.30 |
| ATP-dependent protease subunit HslV | *hslV* | 19.57 | C | 1 | 5.53 |
| hypothetical protein | SA834_19010 | 13.72 | C | 1 | 7.76 |
| DivIVA domain-containing protein | SA834_10700 | 23.51 | C | 1 | 8.78 |
| ABC transporter substrate-binding protein | SA834_05760 | 33.26 | C | 1 | 4.41 |
| 30S ribosomal protein S19 | *rpsS* | 10.62 | C | 1 | 8.70 |
| 30S ribosomal protein S17 | *rpsQ* | 10.17 | C | 1 | 11.49 |

**Table S2. Proteomic analysis of SaEVs (continued).**

| **Protein** | **Gene name** | **MW (kDa)** | **Predicted localization** | **Peptides (95%)** | **%Cov**  **(95)** |
| --- | --- | --- | --- | --- | --- |
| DNA-directed RNA polymerase subunit alpha | *rpoA* | 35.01 | C | 1 | 2.55 |
| transcription termination factor Rho | *rho* | 49.96 | C | 1 | 1.83 |
| type B 50S ribosomal protein L31 | *rpmE* | 9.72 | C | 1 | 16.67 |
| catabolite control protein A | *ccpA* | 36.06 | C | 1 | 2.74 |
| 30S ribosomal protein S4 | *rpsD* | 23.01 | C | 1 | 9.00 |
| acetyl-CoA carboxylase carboxyltransferase subunit beta | *accB_2* | 31.87 | C | 1 | 3.16 |
| hypothetical protein | SA834_14300 | 18.27 | C | 1 | 8.50 |
| chorismate synthase | *aroC* | 42.99 | C | 1 | 2.83 |
| recombinase RecA | *recA* | 37.66 | C | 1 | 3.46 |
| 30S ribosomal protein S12 | *rpsL* | 15.29 | C | 1 | 5.84 |
| Hsp33 family molecular chaperone HslO | SA834_04820 | 31.80 | C | 1 | 3.07 |
| glycosyltransferase family 2 protein | SA834_02530 | 66.13 | C | 1 | 1.40 |
| glycerol-3-phosphate responsive antiterminator | SA834_11760 | 20.45 | C | 1 | 8.89 |
| ribosome assembly RNA-binding protein YhbY | *yhbY* | 11.08 | C | 1 | 14.58 |
| pyridoxal 5'-phosphate synthase glutaminase subunit PdxT | *pdxT* | 20.63 | C | 1 | 6.45 |
| 50S ribosomal protein L25/general stress protein Ctc | *rplY* | 23.89 | C | 1 | 3.23 |
| ROK family glucokinase | *glcK* | 35.09 | C | 1 | 2.44 |
| alpha-ketoacid dehydrogenase subunit beta | SA834_14660 | 36.01 | C | 1 | 5.20 |
| 50S ribosomal protein L23 | *rplW* | 10.61 | C | 1 | 10.99 |

**Table S2. Proteomic analysis of SaEVs (continued).**

| **Protein** | **Gene name** | **MW (kDa)** | **Predicted localization** | **Peptides (95%)** | **%Cov**  **(95)** |
| --- | --- | --- | --- | --- | --- |
| fumarylacetoacetate hydrolase family protein | SA834_08420 | 33.11 | C | 1 | 3.33 |
| NAD-dependent malic enzyme 4 | SA834_16480 | 44.25 | C | 1 | 4.40 |
| alpha/beta hydrolase | SA834_22930 | 35.54 | C | 1 | 3.60 |
| glutamine-hydrolyzing GMP synthase | *guaA* | 58.20 | C | 1 | 1.56 |
| class 1b ribonucleoside-diphosphate reductase subunit beta | *nrdF* | 37.51 | C | 1 | 4.33 |
| orotate phosphoribosyltransferase | *pyrE* | 22.06 | C | 1 | 3.94 |
| DUF47 domain-containing protein | SA834_06310 | 23.74 | C | 1 | 6.83 |
| hypothetical protein | SA834_25380 | 10.70 | C | 1 | 14.29 |
| metal ABC transporter substrate-binding protein | SA834_06000 | 34.74 | CM | 18 | 42.07 |
| MAP domain-containing protein | SA834_18830 | 53.49 | CM | 15 | 32.49 |
| F0F1 ATP synthase subunit beta | *atpD* | 51.40 | CM | 14 | 39.79 |
| metal ABC transporter ATP-binding protein | SA834_06020 | 28.02 | CM | 10 | 41.30 |
| F0F1 ATP synthase subunit B | *atpF* | 19.54 | CM | 10 | 35.26 |
| ATP-dependent metallopeptidase FtsH/Yme1/Tma family protein | SA834_04810 | 77.81 | CM | 6 | 8.32 |
| ATP-binding cassette domain-containing protein | SA834_24600 | 25.76 | CM | 6 | 35.50 |
| amino acid ABC transporter ATP-binding protein | SA834_23530 | 27.24 | CM | 6 | 31.28 |
| **PBP2a family beta-lactam-resistant peptidoglycan transpeptidase MecA** | *mecA_1* | 76.09 | CM | 5 | 5.69 |
| DNA polymerase III subunit beta | *dnaN* | 41.91 | CM | 5 | 13.26 |
| HlyD family secretion protein | SA834_22960 | 23.01 | CM | 5 | 25.12 |
| penicillin-binding protein | SA834_13320 | 80.43 | CM | 5 | 10.45 |

**Table S2. Proteomic analysis of SaEVs (continued).**

| **Protein** | **Gene name** | **MW (kDa)** | **Predicted localization** | **Peptides (95%)** | **%Cov**  **(95)** |
| --- | --- | --- | --- | --- | --- |
| zinc metallopeptidase | SA834_13420 | 25.25 | CM | 5 | 15.52 |
| foldase | SA834_17840 | 35.64 | CM | 4 | 10.31 |
| preprotein translocase subunit YajC | *yajC* | 9.67 | CM | 4 | 22.09 |
| PTS fructose transporter subunit IIC | SA834_06680 | 68.80 | CM | 3 | 4.76 |
| beta-ketoacyl-ACP synthase II | *fabF* | 43.73 | CM | 3 | 7.97 |
| thiol-disulfide oxidoreductase DCC family protein | SA834_20870 | 16.25 | CM | 3 | 18.25 |
| YneF family protein | SA834_12240 | 9.32 | CM | 3 | 13.75 |
| trypsin-like serine protease | SA834_16740 | 45.80 | CM | 3 | 8.49 |
| enoyl-ACP reductase FabI | *fabI* | 27.99 | CM | 3 | 16.80 |
| DoxX family protein | SA834_06750 | 17.67 | CM | 2 | 10.19 |
| alkyl hydroperoxide reductase subunit F | *ahpF* | 54.71 | CM | 2 | 4.73 |
| hypothetical protein | SA834_18690 | 6.56 | CM | 2 | 28.07 |
| preprotein translocase subunit SecG | *secG* | 8.40 | CM | 2 | 33.77 |
| pyruvate oxidase | SA834_24870 | 63.80 | CM | 2 | 3.97 |
| undecaprenyldiphospho-muramoylpentapeptide beta-N-acetylglucosaminyltransferase | SA834_13000 | 39.73 | CM | 2 | 4.49 |
| MetQ/NlpA family ABC transporter substrate-binding protein | SA834_07840 | 30.34 | CM | 1 | 3.30 |
| PTS glucose EIICBA component | SA834_01830 | 73.96 | CM | 1 | 1.17 |
| amino acid ABC transporter permease | SA834_23540 | 26.44 | CM | 1 | 3.35 |
| S41 family peptidase | SA834_13020 | 55.25 | CM | 1 | 1.61 |

**Table S2. Proteomic analysis of SaEVs (continued).**

| **Protein** | **Gene name** | **MW (kDa)** | **Predicted localization** | **Peptides (95%)** | **%Cov**  **(95)** |
| --- | --- | --- | --- | --- | --- |
| ABC transporter substrate-binding protein | SA834_21200 | 36.59 | CM | 1 | 3.67 |
| quinone-dependent dihydroorotate dehydrogenase | SA834_25370 | 39.57 | CM | 1 | 2.82 |
| phenol-soluble modulin export ABC transporter ATP-binding protein PmtC | *pmtC* | 32.94 | CM | 1 | 2.76 |
| DUF2179 domain-containing protein | SA834_18560 | 22.95 | CM | 1 | 4.50 |
| YggT family protein | SA834_10680 | 11.25 | CM | 1 | 10.42 |
| acetolactate synthase AlsS | *alsS* | 61.17 | CM | 1 | 2.35 |
| malate dehydrogenase (quinone) | *mqo_2* | 56.00 | CW | 17 | 36.14 |
| signal peptidase I | *lepB_2* | 21.69 | CW | 8 | 32.46 |
| **bi-component leukocidin LukGH subunit G** | *lukG* | 38.66 | E | 9 | 28.40 |
| **bi-component leukocidin LukGH subunit H** | *lukH* | 40.46 | E | 7 | 20.51 |
| **staphylocoagulase** | *coa* | 68.72 | E | 6 | 7.45 |
| YSIRK domain-containing triacylglycerol lipase Lip2/Geh | *lip2* | 76.54 | E | 6 | 11.72 |
| lytic transglycosylase IsaA | *isaA* | 24.20 | E | 5 | 18.88 |
| **fibrinogen-binding protein** | SA834_10340 | 18.79 | E | 2 | 11.52 |
| **bifunctional autolysin** | SA834_09310 | 136.75 | E | 2 | 2.00 |
| **beta-channel forming cytolysin** | SA834_10390 | 35.98 | E | 2 | 9.09 |
| complement convertase inhibitor Ecb | *ecb* | 12.56 | E | 2 | 6.42 |
| **bi-component gamma-hemolysin HlgAB subunit A** | *hlgA* | 36.38 | E | 1 | 2.49 |
| **bi-component gamma-hemolysin HlgCB subunit C** | *hlgC* | 35.58 | E | 1 | 2.54 |

**Table S2. Proteomic analysis of SaEVs (continued).**

| **Protein** | **Gene name** | **MW (kDa)** | **Predicted localization** | **Peptides (95%)** | **%Cov**  **(95)** |
| --- | --- | --- | --- | --- | --- |
| phage major capsid protein | SA834_19080 | 42.15 | UNK | 29 | 58.27 |
| **5'-nucleotidase, lipoprotein e(P4) family** | SA834_03030 | 33.35 | UNK | 27 | 28.72 |
| **immunoglobulin-binding protein Sbi** | *sbi* | 50.04 | UNK | 19 | 38.76 |
| F0F1 ATP synthase subunit gamma | *atpG* | 32.10 | UNK | 6 | 27.43 |
| NERD domain-containing protein | SA834_17310 | 35.02 | UNK | 6 | 28.15 |
| hypothetical protein | SA834_05800 | 18.59 | UNK | 6 | 36.31 |
| 50S ribosomal protein L13 | *rplM* | 16.33 | UNK | 4 | 30.34 |
| DM13 domain-containing protein | SA834_06760 | 16.05 | UNK | 3 | 13.70 |
| YbbR-like domain-containing protein | SA834_21060 | 34.62 | UNK | 3 | 10.32 |
| phage tail protein | SA834_19020 | 23.81 | UNK | 2 | 13.62 |
| 50S ribosomal protein L15 | *rplO* | 15.60 | UNK | 2 | 20.55 |
| 50S ribosomal protein L4 | *rplD* | 22.46 | UNK | 2 | 10.14 |
| hypothetical protein | SA834_02760 | 57.87 | UNK | 2 | 3.75 |
| transporter substrate-binding domain-containing protein | SA834_23550 | 28.93 | UNK | 2 | 10.04 |
| demethylmenaquinone methyltransferase | SA834_13520 | 27.42 | UNK | 2 | 8.71 |
| **beta-class phenol-soluble modulin** | SA834_10510 | 4.50 | UNK | 2 | 61.36 |
| FKLRK protein | SA834_04060 | 55.5 | UNK | 1 | 1.41 |
| transketolase | *tkt* | 72.25 | UNK | 1 | 1.06 |
| 50S ribosomal protein L21 | *rplU* | 11.33 | UNK | 1 | 9.80 |
| **delta-lysin family phenol-soluble modulin** | *hld* | 5.14 | UNK | 1 | 17.78 |
| DUF4889 domain-containing protein | SA834_23240 | 13.34 | UNK | 1 | 6.78 |

**Table S2. Proteomic analysis of SaEVs (continued).**

| **Protein** | **Gene name** | **MW (kDa)** | **Predicted localization** | **Peptides (95%)** | **%Cov**  **(95)** |
| --- | --- | --- | --- | --- | --- |
| Asp23/Gls24 family envelope stress response protein | SA834_21250 | 19.19 | UNK | 1 | 6.51 |
| HIT family protein | *hit* | 15.94 | UNK | 1 | 7.14 |

Peptides (95%): Number of peptides with 95% confidence to the identified protein.

%Cov (95): Percentage of the protein sequence covered by peptides with 95% confidence.

C: Cytoplasmic; CM: Cell membrane; CW: Cell wall; E: Extracellular; UNK: Unknown.

Virulence factors, drug resistance-related proteins and virulence-related proteins are shown in bold type.


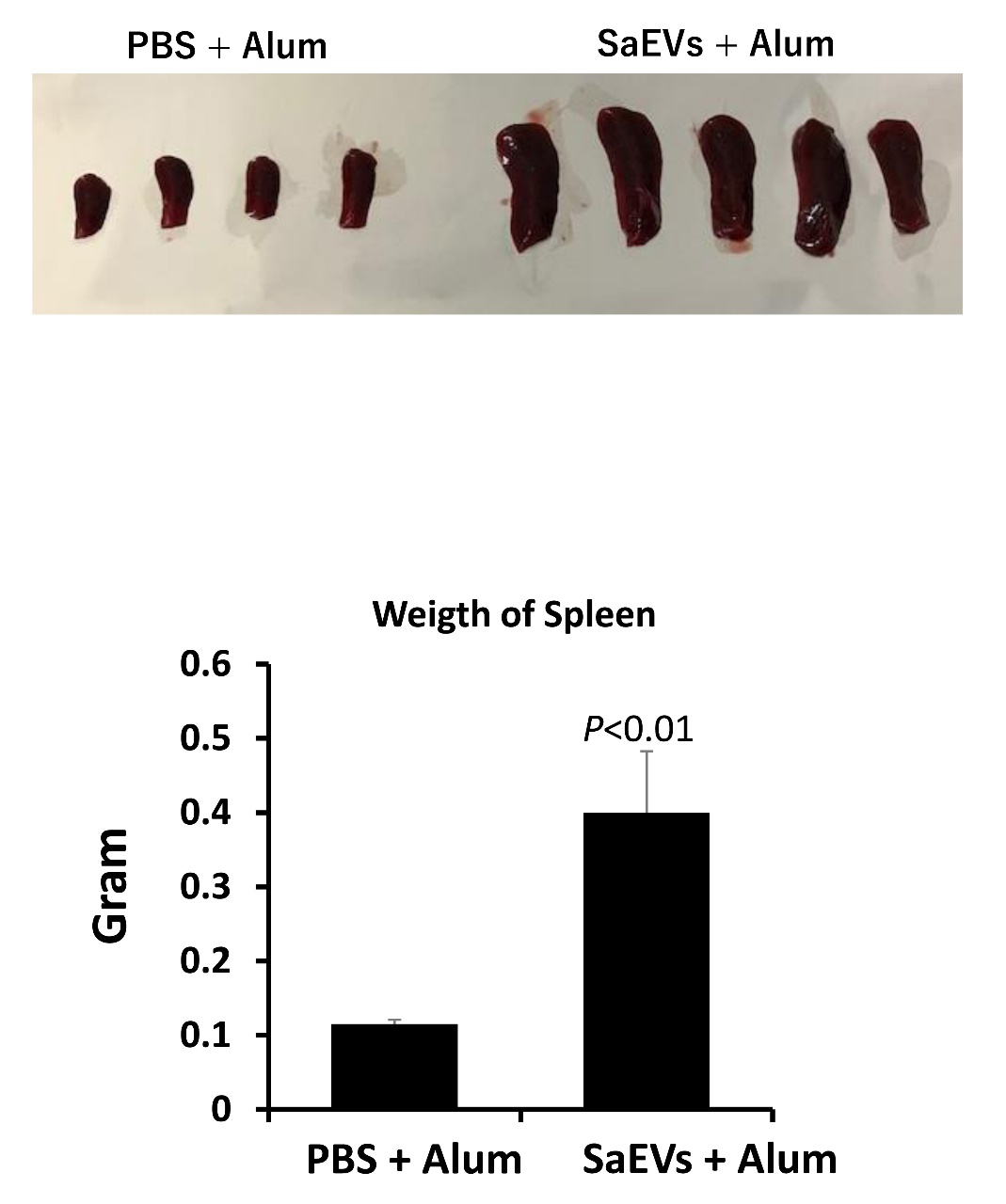


**Figure S6. Weight of spleen collected from SaEV-immunized mice.** Mice were immunized with SaEVs + Alum twice on Day 0 and Day 14. After second immunization for 5 d, spleen were collected and weighed. *P* value was calculated using student *t*-test.


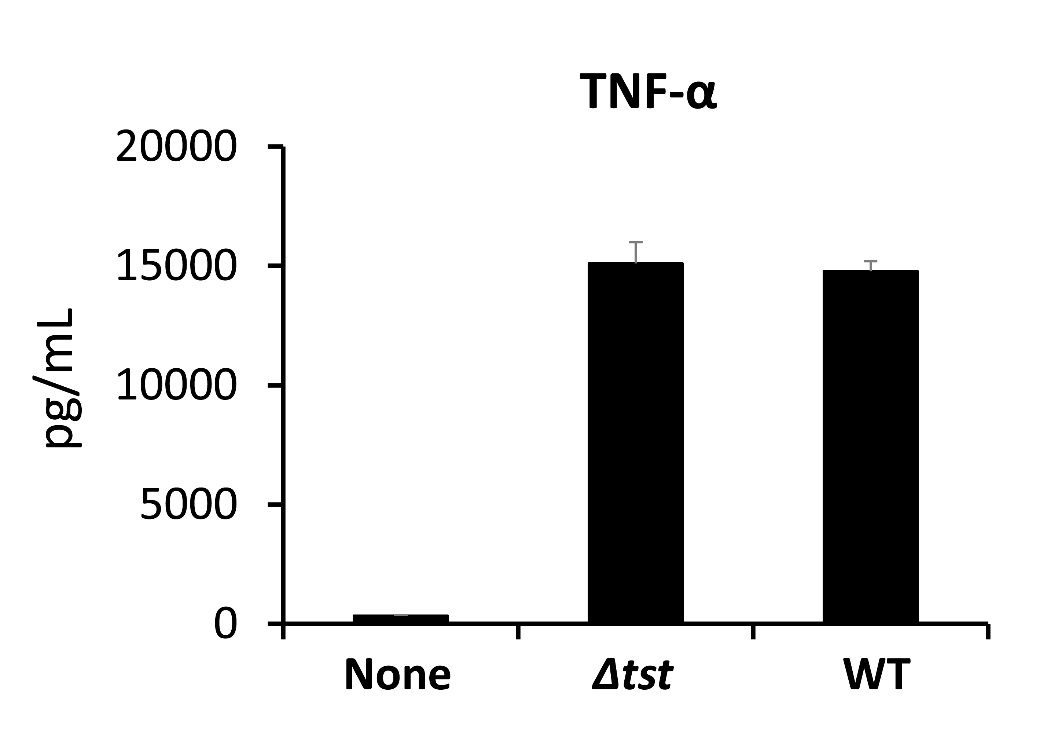


**Figure S7. SaEVs isolated from TSST-1-deficient mutant of MRSA 834 (*Δtst*) stimulated the comparable TNF-α production to the SaEVs isolated from the wild type (WT).** RAW264.7 cells were prepared and adjusted to 2 × 10^6^ cells/mL. They were incubated with 5 μg/mL SaEVs for 72 h. The production of TNF-α in the culture supernatants was determined by ELISA (n=4 from 2-independent experiments).
